# Effect of the Addition of Yellow Mealworm (*Tenebrio molitor*) on the Physicochemical, Antioxidative, and Sensory Properties of Oatmeal Cookies

**DOI:** 10.3390/foods13193166

**Published:** 2024-10-05

**Authors:** Anna Draszanowska, Lidia Kurp, Małgorzata Starowicz, Beata Paszczyk, Marta Czarnowska-Kujawska, Magdalena Anna Olszewska

**Affiliations:** 1 Department of Human Nutrition, The Faculty of Food Science, University of Warmia and Mazury in Olsztyn, Słoneczna 45f, 10-718 Olsztyn, Poland; 2 Department of Chemistry and Biodynamics of Food, Institute of Animal Reproduction and Food Research of Polish Academy of Sciences, Juliana Tuwima 10, 10-748 Olsztyn, Poland; m.starowicz@pan.olsztyn.pl; 3 Department of Commodity Science and Food Analysis, The Faculty of Food Science, University of Warmia and Mazury in Olsztyn, Plac Cieszyński 1, 10-726 Olsztyn, Poland; paszczyk@uwm.edu.pl (B.P.); marta.czarnowska@uwm.edu.pl (M.C.-K.); 4 Department of Food Microbiology, Meat Technology and Chemistry, The Faculty of Food Science, University of Warmia and Mazury in Olsztyn, Plac Cieszyński 1, 10-726 Olsztyn, Poland; magdalena.olszewska@uwm.edu.pl

**Keywords:** oatmeal cookies, yellow mealworm, physicochemical properties, antioxidative properties, texture, sensory analysis

## Abstract

Edible insects are receiving increased attention as a new food source, although research on their implementation in confectionary products remains scarce. The study analyzed the chemical composition, physical parameters, antioxidative, and sensory characteristics of oatmeal cookies reformulated with yellow mealworm larvae (*Tenebrio molitor* L.; TM) at 0% (TM0), 10% (TM10), and 30% (TM30). The inclusion of TM in the cookie recipe increased the protein and fat content, improved the ratio of n-6/n-3 acids, and raised oleic acid levels while reducing palmitic acid. Oatmeal cookies were rich in K and P, and including TM significantly increased the content of most minerals, except for Mn and Na. The cookies held significant antioxidant capacity that increased as the concentration of TM increased due to hydrophilic antioxidants. Although lightness decreased with the increase in mealworm substitution, the yellowness, chroma, and hue angle remained similar for TM0 and TM10. The TM30 cookies were significantly darker and softer, which was further confirmed by panelists. The cookie formulation effectively masked the taste and smell of TM since there were no evident differences between the control and TM10 cookies. Cookies with TM30 received high enough ratings to be considered attractive if differentiated sensory characteristics are desired.

## 1. Introduction

Insects that are fit for human consumption are alternative food sources with high nutritional potential and a high content of bioactive compounds [1,2]. It has been estimated that globally, at least 2 billion people rely on insects as part of their diet, and consumer interest in insect-based foods is gradually increasing [3,4]. According to Jongema [5], over 2100 insect species worldwide have been consumed, such as mealworms, crickets, and silkworm pupae. The growing demand for affordable, high-protein, and/or low-fat food options, coupled with evolving dietary preferences, is expected to drive the market for edible insects. The global edible insect market exceeded USD 112 million in 2019 and is projected to grow at a compound annual growth rate of over 47% from 2019 to 2026 (Global Market Insights Inc.: Selbyville, DE, USA, 2020) [6]. One of the most promising insect species for industrial use and large-scale commercial production is the yellow mealworm, *Tenebrio molitor* L. [7]. Additionally, this is the first insect species approved by the EU for use as human food, and its larvae can be commercialized in frozen, dried, or powdered forms [8].

Unfortunately, entomophagy is still unpopular in Western countries and is often associated with disgust and neophobia [9]. Processing insects can help change the attitudes of people who hold negative views towards consuming insects. Mealing edible insects presents an appealing option, as this approach effectively removes any visual associations with insects. Insect flours have gained attention with different possibilities for inclusion in bakery products, such as in bread formulations [10], muffins [2], cakes [11], pasta [12], and snacks [13]. The inclusion of insect flour in cereal-based products enhances their nutritional value by providing high levels of protein, dietary fiber, unsaturated lipids, and minerals [2]. They have been incorporated into bakery products primarily to increase protein levels and offer protein support, particularly in gluten-free products, or serve as novel ingredients with distinct sensory attributes [14].

Mealworms are of particular interest, with a complete nutritional profile comprising 52.35% protein, 24.70% fat, 2.20% carbohydrates, and 3.62% ash content [15]. They are rich in oleic acid, α-linolenic acid, and linoleic acid, indicating their fairly high-fat content with suitable fatty acid composition [16]. In the fatty acid profile, the highest concentration of desirable oleic acid was obtained for mealworms with a much lower ratio of n-6/n-3 acids compared to buffalo worms or crickets [9]. Moreover, mealworms were reported to contain bioactive compounds that offer various benefits to humans, including antioxidant, antidiabetic, antihypertensive, and anti-inflammatory properties [17,18,19,20].

Based solely on their composition, mealworms could be crucial for enhancing foods that are rich in flour and fat. The incorporation of mealworms into cereal-based products has shown promising results so far. Cozmuta et al. [21] demonstrated that the addition of yellow mealworm powder at 10% to bread increased the bio-accessible fractions of Na, K, Ca, Mg, P, Fe, Zn, Mn, and Li compared to conventional bread made with white flour. Zielińska et al. [2] reported that muffins fortified with yellow mealworm flour at 10% had increased total phenolic content and antioxidant activity compared to control muffins made entirely of wheat flour. This suggests that using yellow mealworms may help to alleviate the nutritional imbalance of such products. However, the inclusion of insects without negatively altering the technological and sensory parameters is challenging and possible to a certain level. For example, Kowalski et al. [9] reported on bread formulated with mealworm flour, resulting in a darker color and distinct smell. This negatively impacted product acceptability, with the addition considered unacceptable above 10%.

Thus, further studies should prioritize enhancing consumer acceptance of edible insect-enriched products by adjusting food compositions or masking off-flavors. The use of traditional ingredients, spices, and regional flavors can enhance product acceptance by improving sensory characteristics such as taste and aroma [14]. Therefore, we further delve into the design of such a product with yellow mealworm larvae (*Tenebrio molitor* L.). This study unravels previously unexplored properties of oatmeal cookies reformulated with a meal of dried mealworms. For that, the oat flakes and whole wheat flour were substituted with the meal at 10 and 30%. In addition, the cookie recipe included ingredients like spices, bananas, or honey, with anticipation of making this mealworm-enriched product more attractive. We are studying how using the meal can affect the physicochemical, textural, antioxidative, color, and sensory characteristics of confectionary products.

## 2. Materials and Methods

### 2.1. Materials and Sample Preparation

**Figure 1:**
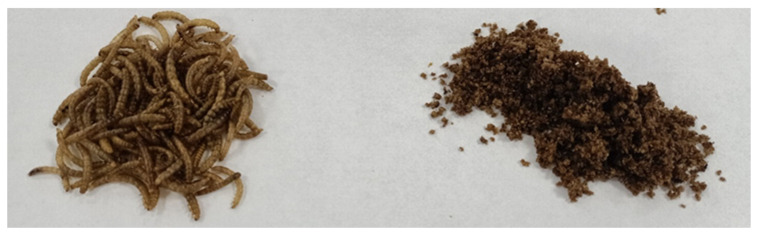
Dried yellow mealworms before (**left**) and after (**right**) milling.

**Figure 2:**
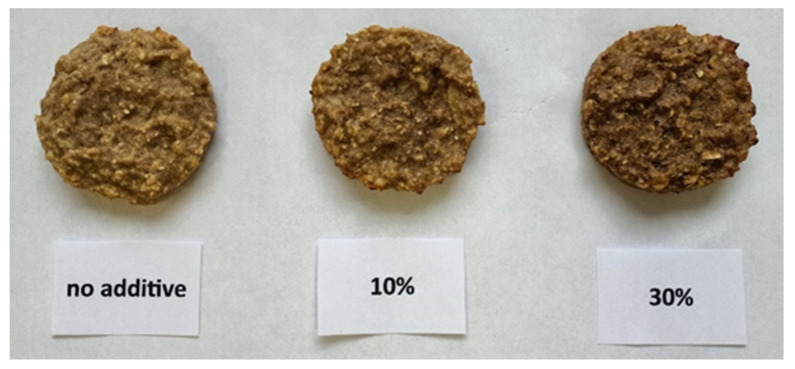
Oatmeal cookies prepared with varying levels of dried mealworms: 0% (no additive), 10%, and 30%.

The ingredients for oatmeal cookies were purchased at a local discount store in Olsztyn, Poland. Dried yellow mealworm (*Tenebrio molitor*) larvae were obtained from Ovad Sp z o.o. in Olsztyn, Poland. The dried mealworm grubs were ground using a multifunctional Robot Coupe^®^ (R 301 ultra D, Vincennes, France) (Figure 1). The first step involved creating oat flake-flour mixes, where oat flakes and whole wheat flour were replaced with dried mealworm meal at levels of 0% (control), 10% (TM10), and 30% (TM30) based on weight ratios presented in Table 1. All ingredients, including shredded apples and crushed bananas, were then combined using a Kenwood planetary robot (KWL90.244SI, Havant, UK). Cookies were formed with a diameter of Ø50 mm and a thickness of approximately 10 mm and baked in a Rational convection oven (SCC WE 101, Landsberg, Germany) with forced airflow at 170 °C for 15 min. After baking, the cookies were cooled down to room temperature in the dark, and part of them were ground in the Robot Coupe^®^ before further analyses. The three types of baked cookies are shown in Figure 2.

### 2.2. Proximate Composition

The oatmeal cookies were analyzed using the AOAC (Association of Official Analytical Chemists) (2005) procedures. Moisture content in samples was determined by drying to constant weight at 105 °C according to AOAC procedure No. 934.01 [22] using a forced draught laboratory oven (UF55, Memmert, Schwabach, Germany). Crude protein content was determined using the Kjeldahl method No. 2001.11 [23]. The process of sample mineralization was carried out using a Kjeldahl digestion system, SpeedDigester K-436 (BÜCHI Labortechnik AG, Flawil, Switzerland), and the distillation process was conducted using the Distillation Unit K-355 (BÜCHI Labortechnik AG, Flawil, Switzerland). Crude fat content was determined using the Soxhlet method No. 991.36 [24] with pure petroleum ether 40/60 p.a. No. 945.16 [25]. The extraction was carried out in Extraction Unit E-816 (BÜCHI Labortechnik AG, Flawil, Switzerland).

### 2.3. Minerals

#### 2.3.1. Reagents and Materials

Hydrated lanthanum chloride (LaCl_3_•7H_2_O); (CAS no. 10025-84-0), used in mineral content determination, was purchased from Merck (Darmstadt, Germany), while ammonium molybdate VI (CAS no. 12054-85-2), sodium sulfate IV (CAS no. 7757-82-6), and hydroquinone (CAS no. 123-31-9) were purchased from “POCH” S. A. (Gliwice, Poland). Other chemicals used in the experiments were at least of analytical grade and were purchased from Merck (Darmstadt, Germany) and “POCH” S.A. (Gliwice, Poland). Folin phenol’s reagent (CAS no. 47641), gallic acid (CAS no. 149-91-7), (±)-6-hydroxy-2,5,7,8-tetramethylchromane-2-carboxylic acid (Trolox); (CAS no. 53188-07-1), sodium bicarbonate (CAS no. 144-55-8) were ordered from Sigma Aldrich Chemical Co. (St. Louis, MO, USA), methanol from Fisher Chemicals (Pittsburgh, PA, USA); (CAS no. 67-56-1), and PCL kits of ACW and ACL from Analytic Jena (Jena, Germany).

#### 2.3.2. Determination of Minerals

Individual elements were determined, as described previously by Klepacka et al. [26] and Czarnowska-Kujawska et al. [27]. Sodium (Na) and potassium (K) were determined using the acetylene-air flame emission technique (an atomic absorption spectrometer, Thermo iCE 3000 Series, Waltham, MA, USA), equipped with a deuterium lamp as a background correction, and appropriate cathode lamps. Determinations of copper (Cu), manganese (Mn), iron (Fe), zinc (Zn), magnesium (Mg), and calcium (Ca) were achieved using a flame atomic absorption spectrometer (Thermo iCE 3000 Series; Madison, WI, USA). The device parameters (air, acetylene, optics, and electronics) were adjusted to achieve maximum absorption for each element. Measurements were carried out at the following wavelengths: Na (589.0), K (766.5 nm), Cu (324.8 nm), Mn (279.5 nm), Fe (248.3 nm), Zn (213.9 nm), Mg (285.2 nm), and Ca (422.7 nm). Phosphorus (P) determination was carried out by colorimetric method with ammonium molybdate, sodium (IV) sulfate, and hydroquinone. Ammonium molybdate (VI) was converted to phosphomolybdates, which were then reduced to phosphomolybdenum blue using sodium (IV) sulfate and hydroquinone. The analysis was carried out using a VIS 6000 spectrophotometer (KRÜSS-OPTRONIC, Hamburg, Germany) set at 610 nm.

### 2.4. Determination of Total Phenolic Content (TPC) and Antioxidant Activity

#### 2.4.1. Extract Preparation

1 mL of 80% methanol solution was added to 100 mg of the sample. Then, the solution was extracted with an ultrasound-assisted method described in detail by Starowicz et al. [28]. Each mixture was vortexed for 30 s, sonicated for 30 s, and finally centrifuged for 5 min (5000× *g*, 4 °C). This step was repeated 5 times. Each time, the residue was resuspended with 1 mL of fresh 80% methanol. The supernatants were combined and collected in a 5-mL volumetric flask. Three independent extractions were carried out for each sample. Extracts were stored in the freezer under −80 °C before further analysis of the TPC and the antioxidant activity.

#### 2.4.2. Total Phenolic Content (TPC)

The TPC method was performed in a microplate reader (Infinite M1000, Tecan, Switzerland), according to Starowicz et al. [28]. Briefly, 0.25 mL of the extract (with a concentration of 20 mg/mL) was mixed with 0.25 mL of the Folin’s phenol reagent, 0.5 mL of saturated sodium carbonate (Na_2_CO_3_), and 4 mL of water. The Folin’s phenol reagent was previously diluted with distilled water (1:1, *v/v*). The obtained mixture was incubated at room temperature (21 °C) for 25 min and centrifuged at 2000× *g* for 10 min. The absorbance of the resulting supernatant was measured at 725 nm. The calibration curve was determined for the gallic acid standard in its concentration range of 0.01–0.70 mg/mL (y = 1.7499x + 0.0321; R^2^ = 0.999). Therefore, the TPC results have been presented as mg gallic acid equivalents per 100 g of dry matter (d. m.).

#### 2.4.3. Antioxidant Activity

Antioxidant activity was measured using the photochemiluminescence method (PCL) with PHOTOCHEM**^®^** apparatus (Analytic Jena, Jena, Germany). The ACW (Antioxidant Capacity of Water-soluble substance) and ACL (Antioxidant Capacity of Liposoluble substance) protocols were employed to measure extracts scavenging activity against superoxide anion radicals (O_2_^•−^). The luminal reagent, Trolox stock, and working solutions were prepared according to the producer’s procedure. Extracts were added with the concentration that ensured the generated luminescence in the range limits of the standard curve. In both modes, Trolox was used as a standard in the range of 0.25–1.00 nmol (R^2^ = 0.9991 in ACW; R^2^ = 0.9956 in ACL). To validate the measurement, typically two blanks and a set of diluted Trolox were run as recommended in the PCL protocol. The obtained results were expressed as µmol Trolox equivalent (TE)/100 g d. m.

### 2.5. Determination of Fatty Acid Profile (FAs)

#### 2.5.1. Fat Extraction

The fat from the cookies was extracted using the Folch method [29]. Briefly, 10 g of the samples (0.01 g) were homogenized with 100 mL of methanol using an IKA Ultra-Turrax**^®^** T18 digital homogenizer (Poland). Then, 100 mL of chloroform was added, and the mixture was filtered. The solid residue was then mixed in 100 mL of chloroform and methanol (2:1 *v/v*) and re-homogenized. After adding sodium chloride at 88%, the mixture was shaken and left overnight. The top layer was removed, and a water:methanol mixture (1:1 *v/v*) was added to the bottom layer, and the washing procedure was repeated. The remaining layer was filtered through anhydrous sodium sulfate, and the solvent was completely evaporated by distillation.

#### 2.5.2. Fatty Acid Profile

The fatty acid methyl esters were prepared from total lipids using the Peisker method with chloroform:methanol:sulphuric acid (100:100:1 *v/v*) as described by Żegarska et al. [30]. The fatty acids of methyl esters of each sample were analyzed using a 6890N chromatograph (Agilent Technologies, Santa Clara, CA, USA) equipped with a flame-ionization detector (FID) under the following conditions: capillary column (dimension 30 m × 0.25 μm with a 0.32 mm internal diameter, liquid phase Stabilwax**^®^** 10 (Supelco, Bellefonte, PA, USA), temperature flame-ionization detector 250 °C, temperature injector 230 °C, column temperature 195 °C, carrier gas—helium with a flow rate 1.5 mL/min. Individual fatty acid methyl esters were identified by comparison to the standard mixture of Supelco 37-component FAME Mix (Supelco, Bellefonte, PA, USA).

### 2.6. Instrumental Texture Analysis

The texture analysis was performed on cookies using a TA.TXplus (Stable Micro Systems Ltd., Godalming, Great Britain) texture analyzer equipped with a 50 kg load cell. The shear force test was performed using a Warner-Bratzler shear blade (Blade Set HDP/BS). The following settings were used to measure hardness: pre-test speed 3.0 mm/s, test speed 0.5 mm/s, post-test speed 10 mm/s, and target-made distance 30 mm.

### 2.7. Color Measurement

The color was measured on cookies using a CR-400 colorimeter (Konica Minolta Sensing Inc., Osaka, Japan) equipped with a standard 2° observer and a D65 illuminator. The device was calibrated using a white ceramic plate provided by the manufacturer. The measurement results were expressed using the CIE L*a*b* color space, in which color is defined by the coordinates L* (light/dark), a* (red/green), and b* (yellow/blue). The color saturation change Chroma (C*), hue angle (h°), browning index (BI), and total color difference (ΔE*) were calculated according to the following equations:

(1) $$C*=\sqrt{a{*}^{2}+b{*}^{2}}$$

h° = arctg(b*/a*) × (360°/2 × 3.14)(2)

BI = 100(x–0.31)/0.172(3)

(4) $$\Delta E*=\sqrt{\Delta L{*}^{2}+\Delta a{*}^{2}+\Delta b{*}^{2}}$$

where:

x = (a* + 1.75L*)/(5.645L* + a* − 0.012b*),

ΔL* = lightness difference,

Δa* = redness difference,

Δb* = yellowness difference.

### 2.8. Sensory Analysis

The evaluation was conducted in the sensory analysis laboratory of the Department of Human Nutrition by a ten-person team of 5 women and 5 men, with an average age ranging from 22 to 40, with proven sensory sensitivity according to EN ISO 8586:2012 [31]. The team was trained for three months (45 h in total), annually practiced, and retrained to keep their skills fresh. Before evaluation, two training sessions familiarized the panelists with the samples represented in the experiment. All panelists had prior experience in evaluating the sensory profiles of insect-based products and provided free, prior informed consent. Three cookies of each type were randomly chosen and placed on white porcelain plates labeled with three-digit codes. The evaluators were given water and bread to cleanse their palates between samples. The panelists were required to score twelve attributes using a linear scale ranging from 0 to 10 points. The sensory attributes were evaluated in the following manner: overall appearance, aroma, and flavor desirability (0-undesirable; 10-highly desirable), intensity of the brown (0-not intensive; 10-very intensive), intensity of mealworm, foreign, and spices aroma and taste (0-undetectable; 10-very intensive), hardness (0-hard; 10-soft), and overall quality (0-poor; 10-very high).

### 2.9. Statistical Analysis

Data analysis was performed using TIBCO**^®^** Statistica™ ver. 13.3 (TIBCO Software Inc., Tulsa, OK, USA), where one-way ANOVA was applied to test the differences between cookies at *p* < 0.05.

## 3. Results and Discussion

### 3.1. Chemical Composition

Edible insects are recognized as a valuable source of protein and fat. The meal of dried mealworms was found to contain 4.02% water, 49.90% protein, and 32.40% fat, which concurs with previous work by Kowalski et al. [9]. Table 2 presents the water, protein, and fat content of each oatmeal cookie formulation. The water content was significantly higher (*p* < 0.05) in cookies without mealworms. The fortification of cookies with dried mealworm resulted in the expected proportional increase in protein and fat content as the addition of the meal increased. The protein content reached 13.66%, and the fat content was 4.16% in cookies with 30% mealworms. The protein content increased by 28.15% and 84.84% for cookies with 10% and 30% mealworms, respectively. The fat content increased by 66.41% and 217.56% for the respective samples. For comparison, Kowalski et al. [11] described the supplementation of sponge cakes with mealworm four at 10% and 20%, showing similar increases in fat content but much less in protein. Ortolá et al. [32] demonstrated a 2.5 times higher increase in protein but a decrease in fat content in biscuits with mealworms at 25%. The current findings indicate that cookies supplemented with 10% TM show similar increases in protein and fat content as Zielińska et al. [2] demonstrated for muffins with 10% TM. However, it is important to note that the original muffins had a fat content of 15.32%.

### 3.2. Minerals Analysis

The mineral composition of each cookie formulation, including the meal of dried mealworms used for the supplementation, is presented in Table 3. The meal had the highest content of Zn (13.45 mg/100 g), followed by Fe, Cu, and Mn, with levels between 3.25 and 0.40 mg/100 g among microelements (Table 3). As for macroelements, the meal was rich in P (1158.35 mg/100 g) and K (964.69 mg/100 g), which aligns with the findings of Ghosh et al. [33] but differs from Cozmuta et al. [21], who reported much lower levels of P. The meal contained lower amounts of Na and Mg, which is consistent with previous reports by Zielińska et al. [15] and Ghosh et al. [33]. However, it had the lowest value of Ca (70.57 mg/100 g) because of the lack of a mineralized skeleton of mealworms. The variations between different studies may be due to differences in sample preparation and drying methods used to obtain dried mealworms [34], as well as the different levels of dietary minerals remaining in the insect gut [21]. The weight of an insect’s gastrointestinal tract can constitute a significant proportion of its total body mass, thereby directly influencing the mineral content of the insect [35]. This underscores the pivotal role of the insect’s diet in determining its mineral composition.

The inclusion of mealworms in cookies has confirmed that mealworm-enriched products can serve as a rich source of minerals (Table 3). Cookies without TM had the highest levels of P (286.93 mg/100 g), Na (275.99 mg/100 g), and K (241.62 mg/100 g), and the lowest levels of Cu (0.19 mg/100 g) and Mn (0.91 mg/100 g). Adding TM to the cookies led to a significant (*p* < 0.05) increase in most of the tested mineral levels, except for Mn and Na, where significant (*p* < 0.05) decreases were observed. In contrast, [21] found that bread enriched with 10% yellow mealworm powder showed no change in Na content and more than a twofold increase in Mn content compared to bread made with 100% white wheat flour.

The addition of 30% TM to the cookies resulted in a twofold increase in Cu and Zn contents. Meanwhile, Fe content increased by 9.5% and 13% in TM10 and TM30, respectively. The lowest increase was observed for Ca, which did not exceed 3% in both TM additions. Magnesium content increased by 18% and 41% in TM10 and TM30 samples, respectively. As for the most abundant minerals, P and K increased by 20% and 17%, respectively, in TM10 samples and 34% and 35% in TM30. Thus, a 100 g serving of cookies containing 30% TM can provide up to 55% of the recommended daily allowance (RDA) for phosphorus in adults [36]. In contrast, [21] reported more than a twofold increase in Fe, even 42%, 90%, and 49% increase in Ca, Mg, and K, respectively, but only a 3.7% increase in P in bread with a 10% yellow mealworm powder. Such variations among products largely result from differences in the mineral content of mealworms used for supplementation.

### 3.3. Antioxidant Properties

The antioxidant properties of oatmeal cookies and the meal used for the supplementation were evaluated based on the determination of total phenolic content (TPC) and the ability to eliminate free radicals by photochemiluminescence (PCL) methods (Table 4). Insects have been found to contain phenolic compounds, mainly by absorbing them from their diet but also through sclerotization, a process in which insects integrate these compounds into the cuticular matrix involving structural proteins and chitin [37]. The TPC in the meal was calculated to be 187.4 mg GAE/100 g d. m., which is comparable to the TPC of certain fruits, such as guava or pomegranate [38]. This TPC, however, was approximately twofold lower than the TPC in mealworm flour reported by Zielińska et al. [2], which could be attributed to the feed given to the insects and variations in the absorption of phenolic compounds from the diet during their developmental stages. The TPC level in control oatmeal cookies (TM0) was calculated at 71.30 mg GAE/100 g, which increased significantly to 95.08 mg GAE/100 g in TM30 samples (*p* < 0.05). For comparison, a previous study by Zielińska et al. [2] found that the control muffins had a total phenolic content of only 6.04 mg GAE/100 g, while the addition of mealworm flour at 10% increased the TPC to 34.11 mg GAE/100 g. Herdeiro et al. [37] also reported an almost fivefold increase in TPC in cereal snacks containing TM. It is important to note that the original oatmeal cookies contained a relatively high concentration of phenolic compounds. This suggests that the ingredients used in the cookie formulation, such as cinnamon, may contribute to the content of phenolics, making the cookies a good source of these valuable constituents. Moreover, it may also be speculated that the higher the TPC of mealworms used for supplementation, the higher the TPC of the cookies and their antioxidant potential.

In the evaluation of the antioxidant capacity, the photochemiluminescence (PCL) methods were utilized to measure water- and lipid-soluble fractions using ACW and ACL kits, respectively. The cookie formulation incorporated a meal with an antioxidant activity of 1190.05 µmol TE/100 g d.m., inferred from the sum of ACW and ACL (PCL = ACW + ACL). This activity was largely attributed to the hydrophobic fraction of antioxidants, which dominated all cookie formulations as well. Although their content in the cookies was not consistent, the ACW assay showed significant increases (*p* < 0.05) in the average content of hydrophilic antioxidants from 103.55 in TM0 to 181.10 µmol TE/100 g d.m. in TM30. As a result, the PCL antioxidant capacity of oatmeal cookies increased in order: TM0 < TM10 < TM30, ultimately reaching 590.75 µmol TE/100 g d.m. In the water-soluble fraction, antioxidants such as flavonoids or amino acids are detected, while in the lipid-soluble fraction, tocopherols, tocotrienols, carotenoids, etc., are measured [39]. The current results show a strong correlation between TPC and ACW, with a Pearson correlation of r = 0.95, suggesting a distinctive contribution of phenolic compounds to the antioxidant properties of cookies with TM. Of note, mealworms, as high-protein products, also represent potential sources of bioactive proteins and peptides. Numerous amino acid sequences derived from the insect have been recognized for their in vitro bioactive properties [40]. Thus, proteins and peptides may also play a role in the antioxidant properties of mealworms and foods fortified with these insects. For comparison, Zielińska and Pankiewicz [41] observed an increase in antioxidant activity in biscuits as the concentration of TM in the recipe increased, demonstrating activities against the DPPH^•^ radical of 2.7 mM TE/100 g in TM flour as opposed to 0.95 mM TE/100 g in biscuits with TM. Similarly, Zielińska et al. (2021) [2] reported levels of 1.21 mM TE/100 g in flour and 0.107–0.223 mM TE/100 g in muffins with mealworms, which was also well correlated with the TPC.

### 3.4. Fatty Acid Composition

The fatty acid profile of the meal used for the supplementation was dominated by oleic acid (C18:1 cis9, n-9), constituting 41.22% (Table 5). In the diet, oleic acid is the most important monounsaturated fatty acid (MUFA) present in such products as olive oil and meat, which has a beneficial effect on health [42]. The second most prevalent fatty acid was linoleic acid (C18:2, n-6) at 28.22%, belonging to the polyunsaturated fatty acids (PUFA), followed by palmitic acid (C16:0) at 17.41%. This concurs with previous work by Kowalski et al. [9] and Cozmuta et al. [43], who also pointed to these three fatty acids as predominant in TM. In the profile of TM meal, palmitoleic acid (C16:1), cis-vaccenic acid (C18:1 cis11), and linolenic acid (C18:3, n-3) were determined at 1.48%, 1.01%, and 2.43%, respectively. Similar amounts to these MUFA/PUFA were quantified for saturated myristic acid (C14:0) and stearic acid (C18:0). In comparison to Kowalski et al. [9] and Kowalski et al. [11], the ratio of n-6/n-3 acids was twofold lower, suggesting a higher amount of n-3 fatty acids in the meal, and the saturated fatty acids (SFA) were lower by 10% compared to Kowalski et al. [9], affecting the fatty acid profile of the cookies.

The current study revealed notable differences in the fatty acid content between cookies with added TM and control oatmeal cookies (TM0). However, oleic acid, linoleic acid, and palmitic acid constituted the majority regardless of cookie formulation (Table 5). MUFA were the predominant fatty acids. In TM0, MUFA accounted for 36.64% and increased as the addition of mealworms increased, reaching 40.92% in TM30, with the most abundant MUFA, oleic acid, increasing incrementally (*p* < 0.05). The inclusion of the meal led to a significant reduction in the SFA from 28.13% in TM0 to 25.47% in TM30, with a consistent decrease in palmitic acid (*p* < 0.05). The TM0 and TM10 had the highest PUFA content at 35.22% and 35.53%, respectively, while the TM30 cookies contained less PUFA at 33.59% (*p* < 0.05). For comparison, Kowalski et al. [11] found significant increases in both MUFA and SFA, alongside a decrease in PUFA from 70.54 to 28.27% in the sponge cakes.

From a nutritional point of view, indicators such as desirable hypocholesterolemic fatty acids (DFA), hypercholesterolemic fatty acids (OFA), atherogenicity index (AI), thrombogenicity index (TI), hypocholesterolemic/hypercholesterolemic ratio (H/H), and the ratio of n-6 to n-3 fatty acids were evaluated. The AI and TI indexes are used as predictors or risk factors for cardiovascular diseases and should be kept low in a healthy daily diet, while the H/H index serves as an indicator of the impact of fatty acids on cholesterol metabolism and should be as high as possible [44]. Moreover, the appropriate balance of PUFA from the n-3 and n-6 families in the diet is crucial [45].

The current data shows that the inclusion of mealworms led to a significant increase in the content of desirable DFA and a notable decrease in the content of undesirable OFA (*p* < 0.05). The ratio of n-6/n-3 acids in oatmeal cookies was considerably high (23.11), but with the addition of the meal, this ratio significantly decreased to 17.78 in TM10 and 14.76 in cookies with 30% insect meal (*p* < 0.05). This aligns with the findings of Cozmuta et al. [43] but contrasts with the results of Kowalski et al. [11], who observed that the n-6/n-3 ratio increased as the concentration of mealworm flour in the sponge cakes increased. Given the high n-6/n-3 ratio in the TM flour utilized by them, the proportion of n-6 and n-3 fatty acids in mealworms used for supplementation of confectionary products could be an important consideration when contemplating dietary habits affecting health benefits from their intake. Moreover, the addition of mealworms to the oatmeal cookies resulted in significant changes in the AI and TI, which were the lowest in cookies with 10% insect meal. Importantly, the H/H index increased incrementally in cookies with added mealworms (*p* < 0.05). Cozmuta et al. [43] and Kowalski et al. [11] showed increases in the indexes of AI and TI and a significant decrease in the H/H index in sponge cakes with added mealworms, suggesting a reduced nutritional quality. Considering the fatty acid profile of oatmeal cookies, adding mealworms has the potential to enhance their nutritional value, dependent not only on mealworms but also on the input value of the supplemented formulation. It should be kept in mind, however, that it is challenging to definitively evaluate the nutritional value of insect lipids. For instance, it has been proposed that the optimal n-6/n-3 ratio should range between 1:1 and 5:1 to maintain a healthy balance (Simopoulos, 2008) [44], suggesting unfavorable ratios for the TM or the cookies. Others emphasize that the total amount of essential fatty acids consumed, including linoleic and linolenic acids, is more important than their n-6/n-3 ratio when considering lifelong dietary habits affecting health [46]. In addition, evidence of the harmfulness of saturated fatty acids exists [47], alongside meta-analyses finding no beneficial effects of reducing SFA intake on, e.g., cardiovascular disease [48].

### 3.5. Texture Analysis

As insects do not contain gluten, the substitution of wheat flour proportionally reduces the gluten content, thereby directly influencing the texture [14]. Oatmeal cookies differed significantly in texture (*p* < 0.05; Table 6). When conducting the breaking test, the maximum force was chosen as a parameter characterizing the texture of the cookies. The highest initial peak was recorded, and this force value was taken as a measure of hardness. Cookies without the addition of insects were characterized by a higher breaking force (34.63 N) than those with the addition of mealworms. The hardness decreased as the addition of mealworms increased, resulting in 30.86 and 23.60 N in TM10 and TM30, respectively (*p* < 0.05). This is probably due to the formation of a weaker gluten network, resulting in a softer consistency of the bakery products [49,50]. Additionally, the decreasing hardness of the cookies with the increasing amount of added mealworms could also be caused by the increase in fat content and the increased share of swollen chitin in the cookies. This result can also be explained by the fact that increasing fat content in the cookies prevented water from evaporating during baking [1]. Although technological challenges related to the texture of these products persist, it is worth mentioning that the decrease in hardness of cookies was not as high as for the sponge cakes [11], crackers [49], and biscuits [50], where the addition of the mealworms did not exceed 20%. This suggests some potential for mealworms to be used at higher levels to reformulate confectionary products like cookies.

### 3.6. Color

In addition to textural features, color is a crucial aspect of bakery products and can significantly influence consumer perception. The CIELab parameters, as well as the total color difference and browning index of oatmeal cookies with added mealworms, are presented in Table 6. The lightness (L*) significantly decreased from 47.59 in TM0 to 42.82 and 34.07 in TM10 and TM30, respectively (*p* < 0.05). Lower L* values with mealworm addition indicate darker cookies due to the mealworm’s darker color, water evaporation during baking, and intensified Maillard reactions from increased protein content in mealworms [41]. A similar effect was reported in shortcake biscuits [41], crackers [49], sponge cakes [11], and muffins [2], with crackers showing the highest 20% reduction of L* for 20% of TM addition. The redness (a*) of the oatmeal cookies increased by only 0.5 and 0.7 in TM10 and TM30, respectively, which was still statistically significant (*p* < 0.05). Meanwhile, the yellowness (b*) decreased by 5.5 but only in TM30 (*p* < 0.05). Much higher increases in the a* parameter were noted by other authors [2,11,41,49], with crackers showing the highest increase again. As for the yellowness, b* increased in sponge cakes [11] and crackers [49], whereas a similar decrease in b* was reported for muffins [2] and biscuits [41], however for 10% of TM incorporation. Chroma C* and the parameter h° are color indicators that reflect the intensity and tone, respectively, taking into account both a* and b* coordinates. The values of C* and h° decreased only in cookies with 30% added mealworms (*p* < 0.05). This aligns with the findings related to muffins [2] but contrasts with the results for crackers [49], which demonstrated an opposing trend.

The total color difference (∆E*) between the control oatmeal cookies and the supplemented cookies was significant because differences above 3 are perceivable by the human eye. However, the ∆E* between TM0 and TM10 was relatively close to it. The ∆E* between TM0 and TM30 was similar to that for muffins [2] and biscuits [41] at 10% TM. In addition, although the browning index (BI) in oatmeal cookies increased significantly by 1.5 times as the addition of TM increased (*p* < 0.05), there were 3 times higher increases in biscuits [41] and sponge cakes [11]. Although the color of bakery products is directly dependent on the colors of the raw materials used, changing their color to a darker color does not necessarily result in a lower consumer evaluation. Insect flour is darker than wheat flour. However, it was suggested that the insect products resemble the color of whole-grain products [51], which our study appeared to confirm. Much lower changes in L*, a*, and BI for oatmeal cookies suggest the potential for mealworms to be used to reformulate them to a satisfactory degree by the consumers.

### 3.7. Sensory Evaluation

The results of the sensory evaluation of oatmeal cookies with the addition of dried mealworms are presented in Table 7. The results indicated that oatmeal cookies with mealworms were well-received, especially cookies supplemented with TM at 10%. In the overall quality sensory assessment, there was no difference between control oatmeal cookies (8.90) and those with 10% TM (8.20). Cookies containing 30% mealworms received significantly lower scores (6.20) (*p* < 0.05), although the scoring was above 5. As the concentration of mealworms increased, the intensity of the brown color significantly increased (*p* < 0.05), which concurs with the sensory analyses of fortified bakery products performed by other authors [2,11,32,52]. However, as suggested by Pauter et al. [51], consumers tend to perceive darker bakery products as healthier and higher in fiber or whole grains. Consequently, this change in color might enhance consumer interest in this type of product. As for the ratings of hardness, the TM0 and TM10 did not differ from each other, whereas TM30 cookies were perceived as softer (*p* < 0.05). Ortolá et al. [32] showed that the higher the mealworm powder concentration, the lower the score in texture and crunchiness in the sensory analysis of biscuits.

The aroma and taste of mealworms slightly intensified as the concentration of mealworms increased, with scores of 0.20–1.10 and 0.30–2.50, respectively (*p* > 0.05). In addition, the panelists rated the intensity of foreign smell and taste as low, ranging from 0.40 to 1.20 and 0.30 to 2.30 (*p* > 0.05). Despite the addition of insects, the intensity of the smell and taste of the spices used in the cookie formulation remained consistent across all samples (*p* > 0.05). The desirability of the smell and taste of oatmeal cookies with TM was rated between 6.30 and 7.70. Notably, TM30 received the lowest scores for both of these attributes. The study by Wendin et al. [52] noted a similar relationship, indicating that the desirability of smell and taste decreased as the amount of mealworm in crisps and pâtés increased. As for the overall liking, Wendin et al. [52] did not observe a significant difference between the control crisps and those with 30% TM as opposed to pâtés. In Xie et al.’s study [50], the addition of 20% mealworm had a significant impact on the overall acceptability of biscuits as compared to lower TM additions. In the research conducted by Çabuk [53], it was determined that the incorporation of 15% mealworm powder resulted in muffins with sensory scores ranging from 6.70 to 8.70 across all evaluated attributes. In another study, the panelists rated the appearance, taste, texture, and overall acceptance of sponge cakes as significantly worse when wheat flour was replaced by 10% and 20% TM [11]. Similarly, muffins with a TM addition level between 2 and 10% decreased all the attributes tested, including color, consistency, smell, taste, and overall acceptability [2].

## 4. Conclusions

The utilization of *Tenebrio molitor* presents a new opportunity for confectionery products, such as oatmeal cookies, to sustainably support the recent trend in the bakery market towards product reformulation. The inclusion of TM in the cookie recipe increased the protein, fat, and mineral contents, improved the n-6/n-3 ratio, reduced the content of palmitic acid, and increased the oleic acid, deserving an important consideration. The cookies had a considerable antioxidant capacity that increased as the concentration of TM increased due to hydrophilic antioxidants. The lightness decreased, but other parameters, such as yellowness, chroma, and hue angle, remained similar for TM0 and TM10. Thus, only TM30 cookies were darker and softer. The study found that adding yellow mealworm to oatmeal cookies, particularly the addition of 10% TM, was well-received by respondents. The ratings from the evaluation panel for cookies with TM30 were sufficiently high to be considered appealing if distinctive sensory characteristics are desired.

## Figures and Tables

**Table 1:** Oatmeal cookies’ formulation and coding.

Ingredient (g)	Formulation and Coding		
	(Ratio of TM *)		
	TM0 (0%)	TM10 (10%)	TM30 (30%)
Dried mealworm	0	38	114
Oat flakes	200	180	140
Whole wheat flour	180	162	126
Baking powder	8	8	8
Cardamom	2	2	2
Cinnamon	2	2	2
Salt	0.5	0.5	0.5
Eggs	112	112	112
Multifloral honey	100	100	100
Apples	300	300	300
Bananas	150	150	150

TM—*Tenebrio molitor.* * The TM ratio is calculated as the total amount of oat flakes, whole wheat flour, and yellow mealworm meal.

**Table 2:** Composition of oatmeal cookies formulated with dried yellow mealworms in terms of water, protein, and fat content.

	Samples	% Water	% Protein	% Fat
Raw material	Meal	4.02	49.90	32.40 *
Cookies	TM0	42.41 ± 0.16 ^b^	7.39 ± 0.08 ^a^	1.31 ± 0.05 ^a^
	TM10	40.38 ± 0.16 ^a^	9.47 ± 0.21 ^b^	2.18 ± 0.19 ^b^
	TM30	40.68 ± 0.13 ^a^	13.66 ± 0.20 ^c^	4.16 ± 0.26 ^c^

TM—*Tenebrio molitor*; Values are expressed as means (n = 3) ± standard deviations. Mean values with different letters (^a^, ^b^, ^c^) in the column are statistically different (*p*-value < 0.05). * provided by the manufacturer.

**Table 3:** Mineral composition in oatmeal cookies formulated with dried yellow mealworms.

		Microelements (mg/100 g)				Macroelements (mg/100 g)				
	Samples	Cu	Mn	Fe	Zn	Mg	Ca	Na	K	P
Raw material	Meal	1.84 ± 0.035	0.40 ± 0.012	3.25 ± 0.047	13.45 ± 0.198	194.58 ± 3.871	70.57 ± 0.442	736.40 ± 2.401	964.69 ± 5.142	1158.35 ± 52.049
Cookies	TM0	0.19 ± 0.004 ^a^	0.91 ± 0.035 ^c^	1.26 ± 0.036 ^a^	1.16 ± 0.026 ^a^	35.99 ± 0.571 ^a^	40.58 ± 0.058 ^a^	275.99 ± 2.179 ^c^	241.62 ± 0.414 ^a^	286.93 ± 3.299 ^a^
	TM10	0.26 ± 0.005 ^b^	0.86 ± 0.050 ^b^	1.38 ± 0.018 ^b^	1.73 ± 0.005 ^b^	42.59 ± 0.881 ^b^	41.45 ± 0.062 ^b^	225.69 ± 2.416 ^a^	282.88 ± 1.141 ^b^	344.37 ± 2.451 ^b^
	TM30	0.38 ± 0.012 ^c^	0.71 ± 0.014 ^a^	1.42 ± 0.026 ^b^	2.57 ± 0.026 ^c^	50.91 ± 0.438 ^c^	41.68 ± 0.072 ^c^	245.19 ± 3.201 ^b^	326.99 ± 3.654 ^c^	384.39 ± 3.525 ^c^

TM—*Tenebrio molitor*; Values are expressed as means (n = 3) ± standard deviation. Mean values with different letters (^a^, ^b^, ^c^) in the column are statistically different (*p*-value < 0.05).

**Table 4:** Total phenolic content (TPC) and antioxidant activity in oatmeal cookies formulated with dried yellow mealworms.

	Samples	TPC	ACW	ACL	PCL
		[mg GAE/ 100 g d.m.]	[µmol TE/ 100 g d.m.]		
Raw material	Meal	187.40 ± 5.96	443.43 ± 9.40	746.61 ± 14.04	1190.05 ± 16.25
Cookies	TM0	71.30 ± 2.26 ^a^	103.55 ± 1.77 ^a^	418.24 ± 17.26 ^a^	521.79 ± 18.29 ^a^
	TM10	76.68 ± 2.53 ^a^	142.05 ± 2.25 ^b^	431.88 ± 6.51 ^a^	573.93 ± 8.18 ^a,b^
	TM30	95.08 ± 2.71 ^b^	181.10 ± 4.16 ^c^	409.65 ± 17.15 ^a^	590.75 ± 21.29 ^b^

TM—*Tenebrio molitor*; GAE—gallic acid equivalent; d.m.—dry matter; TE—Trolox equivalent; PCL—sum of ACW and ACL. The antioxidant capacity of the water- (ACW) and lipid-soluble (ACL) components. Values are expressed as means (n = 3) ± standard deviations. Mean values with different letters (^a^, ^b^, ^c^) in the column are statistically different (*p*-value< 0.05).

**Table 5:** Fatty acid profile of oatmeal cookies formulated with dried yellow mealworms.

	Raw Material		Cookies	
Samples	Meal	TM0	TM10	TM30
Fatty Acids	% of the Total Detected Fatty Acids			
C12:0	0.25 ± 0.04	0.29 ± 0.02 ^c^	0.13 ± 0.01 ^a^	0.19 ± 0.01 ^b^
C14:0	2.58 ± 0.04	0.46 ± 0.03 ^a^	1.06 ± 0.02 ^b^	1.67 ± 0.02 ^c^
C16:0	17.41 ± 0.47	22.28 ± 0.14 ^c^	20.21 ± 0.18 ^b^	18.99 ± 0.03 ^a^
C16:1	1.48 ± 0.05	1.72 ± 0.03 ^b^	1.50 ± 0.02 ^a^	1.69 ± 0.03 ^b^
C17:0	0.24 ± 0.02	0.12 ± 0.01 ^a^	0.17 ± 0.02 ^b^	0.21 ± 0.02 ^c^
C17:1	0.21 ± 0.04	0.11 ± 0.01 ^a^	0.14 ± 0.02 ^b^	0.19 ± 0.01 ^c^
C18:0	3.39 ± 0.02	4.49 ± 0.03 ^c^	4.08 ± 0.02 ^b^	4.00 ± 0.02 ^a^
C18:1 *cis*9 (*n*-9)	41.22 ± 0.18	32.67 ± 0.07 ^a^	34.91 ± 0.03 ^b^	37.41 ± 0.09 ^c^
C18:1 *cis*11	1.01 ± 0.07	1.66 ± 0.01 ^c^	1.36 ± 0.02 ^b^	1.25 ± 0.03 ^a^
C18:2 (*n*-6)	28.22 ± 0.19	32.16 ± 0.04 ^b^	32.42 ± 0.04 ^c^	30.22 ± 0.01 ^a^
C18:3 (*n*-3)	2.43 ± 0.04	1.49 ± 0.01 ^a^	1.95 ± 0.01 ^b^	2.22 ± 0.02 ^c^
C20:0	0.20 ± 0.00	0.09 ± 0.01 ^a^	0.17 ± 0.02 ^b^	0.17 ± 0.01 ^b^
C20:1	0.33 ± 0.05	0.48 ± 0.04 ^c^	0.43 ± 0.03 ^b^	0.37 ± 0.02 ^a^
C20:4 (*n*-6)	N.D.	0.76 ± 0.04 ^c^	0.50 ± 0.01 ^b^	0.39 ± 0.02 ^a^
C22:0	0.47 ± 0.04	0.41 ± 0.03 ^c^	0.32 ± 0.02 ^b^	0.24 ± 0.03 ^a^
C22:5 (*n*-6)	0.53 ± 0.05	0.81 ± 0.04 ^c^	0.66 ± 0.03 ^a^	0.76 ± 0.04 ^b^
ΣSFA	24.53 ± 0.51	28.13 ± 0.10 ^c^	26.10 ± 0.10 ^b^	25.47 ± 0.09 ^a^
ΣMUFA	44.26 ± 0.27	36.64 ± 0.02 ^a^	38.35 ± 0.05 ^b^	40.92 ± 0.07 ^c^
ΣPUFA	31.17 ± 0.23	35.22 ± 0.09 ^b^	35.53 ± 0.05 ^c^	33.59 ± 0.03 ^a^
*n*-6/*n*-3 ratio	12.16 ± 0.11	23.11 ± 0.16 ^c^	17.78 ± 0.18 ^b^	14.76 ± 0.15 ^a^
DFA	75.43 ± 0.43	71.86 ± 0.09 ^a^	73.88 ± 0.10 ^b^	74.51 ± 0.08 ^c^
OFA	21.15 ± 0.52	23.64 ± 0.12 ^c^	22.05 ± 0.12 ^b^	21.47 ± 0.07 ^a^
AI index	0.37 ± 0.01	0.34 ± 0.003 ^b^	0.33 ± 0.002 ^a^	0.35 ± 0.002 ^c^
TI index	0.42 ± 0.01	0.43 ± 0.003 ^b^	0.42 ± 0.003 ^a^	0.43 ± 0.002 ^a,b^
H/H ratio	3.58 ± 0.11	2.95 ± 0.03 ^a^	3.29 ± 0.03 ^b^	3.40 ± 0.01 ^c^

TM—*Tenebrio molitor*; Values are expressed as means (n = 3) ± standard deviation. N.D.—not detected. Mean values with different letters (^a^, ^b^, ^c^) in the row are statistically different (*p*-value < 0.05). SFA—saturated fatty acids; MUFA—monounsaturated fatty acids; PUFA—polyunsaturated fatty acids; DFA—hypocholesterolemic fatty acids (ΣUFA + C18:0); OFA—hypercholesterolemic fatty acids (ΣSFA-C18:0); AI (atherogenic index) = (C12:0+4 × C14:0 + C16:0)/(Σn-6 + Σn-3 + ΣMUFA) and TI (thrombogenic index) = (C14:0 + C16:0 + C18:0)/0.5 × ΣMUFA + 0.5 × Σn-6 + 3 × Σn-3 + (Σn-3/Σn-6) (Ulbricht & Southgate, 1991, Osmari et al., 2011); H/H (hypocholesterolemic/hypercholesterolemic ratio) = (C18:1 + C18:2 + C18:3 + C20:2 + C20:4 + C20:5 + C22:5 + C22:6)/(C12 + C14:0 + C16:0) (Ivanova & Hadzhinikolova 2015).

**Table 6:** Color and texture characteristics of the oatmeal cookies formulated with dried yellow mealworms.

Samples	L*	a*	b*	C*	h°	BI	∆E*	Hardness [N]
TM0	47.59 ± 2.41 ^c^	4.84 ± 0.22 ^a^	21.83 ± 0.87 ^b^	22.36 ± 0.85 ^b^	77.47 ± 0.74 ^b^	7.31 ± 0.48 ^a^	-	34.63 ± 2.57 ^c^
TM10	42.82 ± 2.69 ^b^	5.39 ± 0.36 ^b^	21.58 ± 1.36 ^b^	22.25 ± 1.32 ^b^	75.92 ± 1.26 ^b^	8.99 ± 0.85 ^b^	5.12	30.86 ± 2.59 ^b^
TM30	34.07 ± 2.86 ^a^	5.53 ± 0.35 ^b^	16.31 ± 1.56 ^a^	17.23 ± 1.48 ^a^	71.16 ± 2.44 ^a^	11.45 ± 0.86 ^c^	14.66	23.60 ± 1.95 ^a^

TM—*Tenebrio molitor*; Values are expressed as means (n = 10) ± standard deviation. Mean values with different letters (^a^, ^b^, ^c^) in the column are statistically different (*p*-value < 0.05). Lightness (L*) and color (a*—redness, b*—yellowness). Chroma (C*) and hue (h◦). ∆E—total color difference, BI—browning index.

**Table 7:** The sensory quality of oatmeal cookies formulated with dried yellow mealworms.

Attributes		Samples		
		TM0	TM10	TM30
Overall appearance		8.90 ± 0.74 ^b^	8.20 ± 0.92 ^b^	6.20 ± 1.23 ^a^
Intensity of	brown color	4.60 ± 0.97 ^a^	6.90 ± 0.74 ^b^	9.10 ± 0.88 ^c^
	mealworm aroma	0.20 ± 0.42 ^a^	0.30 ± 0.67 ^a^	1.10 ± 1.29 ^a^
	foreign aroma	0.40 ± 0.70 ^a^	0.50 ± 0.85 ^a^	1.20 ± 1.23 ^a^
	spices aroma	5.00 ± 0.82 ^a^	4.90 ± 0.57 ^a^	4.80 ± 1.99 ^a^
Aroma desirability		7.60 ± 0.97 ^b^	7.60 ± 1.26 ^b^	6.30 ± 0.82 ^a^
Hardness		6.60 ± 1.71 ^a^	6.30 ± 1.06 ^a^	8.30 ± 0.95 ^b^
Intensity of	mealworm taste	0.30 ± 0.67 ^a^	0.70 ± 0.82 ^a^	2.50 ± 1.27 ^b^
	foreign taste	0.30 ± 0.67 ^a^	0.90 ± 0.88 ^a^	2.30 ± 1.16 ^b^
	spices taste	6.00 ± 0.94 ^a^	5.20 ± 0.79 ^a^	5.50 ± 1.27 ^a^
Flavor desirability		7.80 ± 1.32 ^a^	7.70 ± 1.34 ^a^	6.60 ± 1.26 ^a^
Overall quality		7.30 ± 1.16 ^a^	7.10 ± 1.20 ^a^	6.30 ± 1.34 ^a^

TM—*Tenebrio molitor*; Values are expressed as means (n = 10) ± standard deviations. Mean values with different letters (^a^, ^b^, ^c^) in the row are statistically different (*p*-value < 0.05).

## Data Availability

The original contributions presented in the study are included in the article, further inquiries can be directed to the corresponding author.
